# Maslinic acid induces autophagy and ferroptosis via transcriptomic and metabolomic reprogramming in prostate cancer cells

**DOI:** 10.3389/fphar.2024.1453447

**Published:** 2024-11-18

**Authors:** Fen Hu, Yuxi Sun, Yunfeng Zhang, Jiaxin Chen, Yingzi Deng, Yifei Li, Ruobing Li, Juan Zhang, Yongping Liang, Yan Liu, Shuqing Wang, Mi Li, Lina Zhao, Yuwei Liu, Xiaodong Gong, Haifeng Cai, Shouqin Gu

**Affiliations:** ^1^ College of Life Sciences, North China University of Science and Technology, Tangshan, Hebei, China; ^2^ Tangshan Key Laboratory of Agricultural Pathogenic Fungi and Toxins, Department of Life Sciences, Tangshan Normal University, Tangshan, Hebei, China; ^3^ The Second Department of Breast Surgery, Tangshan People’s Hospital, Tangshan, Hebei, China; ^4^ College of Life Sciences, Hebei Agricultural University, Baoding, Hebei, China; ^5^ Hebei Bioinformatics Utilization and Technological Innovation Center for Agricultural Microbes, Hebei Agricultural University, Hebei, China

**Keywords:** maslinic acid, autophagy, transcriptome, metabolome, ferroptosis

## Abstract

Prostate cancer has the second highest incidence among male malignancies. Only a few studies exist on the inhibitory effects of maslinic acid (MA) on prostate cancer. Herein we found that MA inhibits prostate cancer cell proliferation by decreasing CDK2, CDK4, and CDK6 expression and concurrently increasing p27, Rb, p-Rb expression. Further, MA was observed to induce prostate cancer cell autophagy by increasing the expression of p53, p-p53, ULK1, Beclin1, Atg7, and Atg5 and the ratio of LC3-II/I and concurrently decreasing the expression of ERK1/2 and mTOR. In addition, MA induced RM-1 cell ferroptosis by regulating glutathione, glutamate, and oxidized glutathione concentrations, inhibiting *SLC7A11* activity, and downregulating GPX4 expression. Integrated metabolome and transcriptome analysis led to the identification of key pathways (e.g., pathways in cancer and glutathione metabolism). Real-time quantitative PCR confirmed that MA regulates the expression of *ABCA1*, *JUN*, and *NFKBIA*. *In vivo*, we demonstrated that 50 mg/kg MA significantly inhibited the growth of tumors established using RM-1 cells. To summarize, we report that MA inhibits prostate cancer cell growth both *in vitro* and *in vivo* by inducing autophagy and ferroptosis via transcriptomic and metabolomic reprogramming.

## 1 Introduction

The incidence of prostate cancer ranks second among malignant tumors in men globally, and it is the primary cause of cancer-related mortality in males. According to GLOBOCAN 2020 data, 1.4 million new cases and 375,000 deaths were reported worldwide (Ferlay et al., 2021). Surgery, radiation therapy, chemotherapy, and combination therapy are the main and effective clinical treatment options for prostate cancer; however, there is no effective treatment for castration-resistant prostate cancer (CRPC) (Sekhoacha et al., 2022). Chemotherapy often leads to drug resistance in prostate cancer, prompting ongoing research for new, effective drugs.

Pentacyclic triterpenes represent an important class of secondary metabolites found in plants. With broad antitumor activity and no apparent toxicity, they are promising lead compounds for developing new multitargeting antitumor agents. Maslinic acid [MA, C30H48O4, (2alpha, 3beta)-2,3- dihydroxy-olean-12-en-28-oic acid] is an oleanane-type pentacyclic triterpene that exhibits various pharmacological activities, including anticancer (Ooi et al., 2023; Yu et al., 2021), antioxidant (Li et al., 2023b), and anti-inflammatory (Wang et al., 2022) effects. Park et al. (2013) found MA to inhibit the metastatic capacity of DU145 human prostate cancer cells by affecting cell invasion, migration, adhesion, and angiogenesis, a process involving hypoxia-inducible factor-1α signaling. However, in general, only a few studies have reported the effects of MA on prostate cancer.

Ferroptosis is a novel mode of cell death mediated by the accumulation of iron-dependent lipid peroxidation (Jiang et al., 2021). Some approved drugs, such as sorafenib (Li et al., 2023a), artemisinin (Wang et al., 2024), and statins (Sahebkar et al., 2023), can induce ferroptosis and suppress tumor growth (Chen et al., 2021). Tian et al. prepared mitochondria-targeted pentacyclic triterpenoid carbon dots using glycyrrhetinic acid, ursolic acid, and oleanolic acid as precursors, reporting selective cancer cell destruction through ferroptosis, autophagy, and apoptosis (Tian et al., 2023).

Herein we investigated the effects and mechanisms of MA on prostate cancer cell proliferation, autophagy, and ferroptosis. Transcriptomics and metabolomics analyses were performed to determine potential pathways through which MA affects prostate cancer cells, with the aim of identifying new therapeutic strategies for prostate cancer.

## 2 Material and methods

### 2.1 Cell culture

RM-1 (an androgen-insensitive mouse prostate cancer cell line). LNCaP (an androgen-responsive cell line). LNCaP and RM-1 cells (American Type Culture Collection, United States) were cultured in high-glucose Dulbecco’s modified Eagle medium supplemented with 10% fetal bovine serum, penicillin, and streptomycin. All cells were cultured at 37°C and 5% CO_2_. MA (Sigma-Aldrich, China) was prepared in dimethyl sulfoxide (DMSO).

### 2.2 (3-(4,5-Dimethylthiazol-2-yl)-2,5-diphenyltetrazolium bromide) (MTT) assay

After 24 h of MA treatment, MTT assay was performed as previously described (Jain and Grover, 2020).

### 2.3 5-Ethynyl-2′-deoxyuridine (EdU) cell proliferation assay

After 24 h of MA treatment, EdU cell proliferation assay was performed as described previously (Hu et al., 2013).

### 2.4 Western blotting

The following antibodies were used: anti-CDK2 (ab235941), anti-CDK4 (ab68266), anti-CDK6 (ab151247), anti-p27 (ab215434), anti-Rb (ab226979), anti-p-Rb (A23807), anti-LC3B (ab221794), anti-p53 (D291561-0050), anti-p-p53 (A44956), anti-ERK1/2 (ab17942), anti-Atg5 (ab228668), anti-Atg7 (ab133528), anti-ULK1 (ab229909), anti-Beclin1 (ab210498), anti-mTOR (D155337-0025), anti-GPX4 (ab125066), anti-β-actin (ab115777), and goat anti-rabbit IgG H&L (ab6721). At 24 h post-treatment with various concentrations of MA, Western blotting was performed as previously reported (Liu et al., 2013).

### 2.5 Autophagic flux analysis

LNCaP and RM-1 cells were seeded in 96-well culture plates. Upon reaching 80%–90% confluency, they were transfected with 200 ng mRFP-GFP-LC3 plasmid (Changsha Youbao, China) using Lipofectamine 2000 (Invitrogen, United States). At 24 h post-transfection, the cells were treated with different concentrations of MA for 24 h. Autophagic flux was observed under an inverted fluorescent microscope.

### 2.6 Transmission electron microscopy

RM-1 cells were treated with 20 μM MA for 24 h. They were then fixed, dehydrated, stained, and finally observed under a transmission electron microscope as previously described (Tian et al., 2018).

### 2.7 RNA-seq

RM-1 cells were randomly divided into eight groups. Four groups were treated with 20 μM MA for 24 h, while the other four were treated with DMSO (control). Total RNA was extracted from the cells using TRIzol. RNA-seq was performed by Shanghai Applied Protein Technology Biotechnology Co. Ltd. (Shanghai, China) on the Illumina HiSeq sequencing platform. Sequencing libraries were generated and sequenced as described by Wen et al. (2021). Differentially expressed mRNAs were identified based on |log2 (fold-change)| > 1 and *p* < 0.05 using edgeR or DESeq2. Differentially expressed genes (DEGs) were subjected to Kyoto Encyclopedia of Genes and Genomes (KEGG) pathway enrichment analysis.

### 2.8 Metabolome analysis

RM-1 cells were randomly divided into 12 groups. Six groups were treated with 20 μM MA for 24 h, while the remaining were treated with DMSO (control). Collection and dissolution of RM-1 cells and liquid chromatography–tandem mass spectrometry were performed as described by Chen et al. (2020). Significantly differential metabolites were identified based on variable importance in projection score >1 (OPLS-DA model) and *p* < 0.05 (Student’s t-test). KEGG pathway enrichment analysis was performed with GOATOOLS and KOBAS 2.1.1. Integrated transcriptome and metabolome analyses were conducted using iPath 3.0 to profile significantly altered metabolic pathways (Fu et al., 2022). A heatmap was plotted using https://www.bioinformatics.com.cn (last accessed: July 10, 2023).

### 2.9 Real-time quantitative PCR (RT-qPCR)

Total RNA was isolated using TRIzol from RM-1 cells, which were treated with 20 μM MA for 24 h. RT-qPCR was performed as previously described (Hu et al., 2019) using the 2^−△△CT^ method (Livak and Schmittgen, 2001). Primer pairs are shown in Table 1. *GAPDH* was used as an internal control.

**TABLE 1:** Primers for RT-qPCR.

Gene name	Primer (5′-3′)
*SLC7A11*	F: ATT​ATA​GAG​CCA​GCG​AAG​GCT​GA
	R: TGA​CAG​TAC​TCC​ACA​GGC​AG
*ABCA1*	F: GGCAACAAACGAAAGCTC
	R: CTT​AGG​GCA​CAA​TTC​CAC​A
*JUN*	F: GCT​GGA​AGA​AGG​GCT​GTT​G
	R: CAG​ATT​CAA​AGT​TGG​AAG​GAG​AC
*NFKBIA*	F: AGCACAAAGAGAGTCGC
	R: CAGCGTTCATGGTTATGG
*GAPDH*	F: GAG​ATT​ACT​GCT​CTG​GCT​CCT​A
	R: TGG​TCC​AGG​GTT​TCT​TAC​TC

### 2.10 Calcein/PI cell activity assay

RM-1 cells were plated in a 96-well plate and treated with MA with or without Erastin (Beyotime, SC0224) for 24 h. Staining was subsequently performed as per the instructions of the Calcein/PI Cell Viability/Cytotoxicity Assay Kit (Beyotime, C2015S), followed by visualization using a fluorescent microscope.

### 2.11 Intracellular reactive oxygen species (ROS) levels

RM-1 cells were plated in a 96-well plate and treated with MA with or without Erastin for 24 h. Intracellular ROS levels were subsequently determined using the Reactive Oxygen Species Assay Kit (Beyotime, S0033S).

### 2.12 Glutathione (GSH), glutamate (Glu), and oxidized GSH (GSSG) assay

Intracellular GSH, Glu, and GSSG levels were measured using a kit (Solarbio, BC1585 and Beyotime, S0053) according to manufacturer instructions. The values were normalized to protein concentration.

### 2.13 Colony formation assay

RM-1 cells (100 cells/well) were seeded in 6-well culture plates and treated with various concentrations of MA or DMSO for 7 days. The experiment was terminated when colony formation was visible to the naked eye. The cells were washed twice with phosphate-buffered saline and then fixed with 4% cell fixative for 30 min. They were subsequently stained with crystal violet for 15 min in the dark. The cells were then washed with water and photographed.

### 2.14 *In Vivo* model

For the preliminary experiment, 1 × 10^6^ RM-1 cells were subcutaneously injected into 10 five-week-old C57/B6 mice. After the tumors had established (approximately 60 mm^3^), the mice were subcutaneously injected with 25 mg/kg or 50 mg/kg MA every 2 days. Control mice were injected with 20% β-cyclodextrin. Body weight and tumor sizes were recorded. After 19 days, the mice were euthanized, and the tumors were collected for analysis.

### 2.15 Statistical analysis

Values represent mean ± standard deviation. Statistical analysis was conducted using SPSS v16.0. Student’s t-test was applied to compare MA-treated and control cells. *p* < 0.05 indicated statistical significance.

## 3 Results

### 3.1 Inhibition of prostate cancer cell proliferation by MA

**FIGURE 1:**
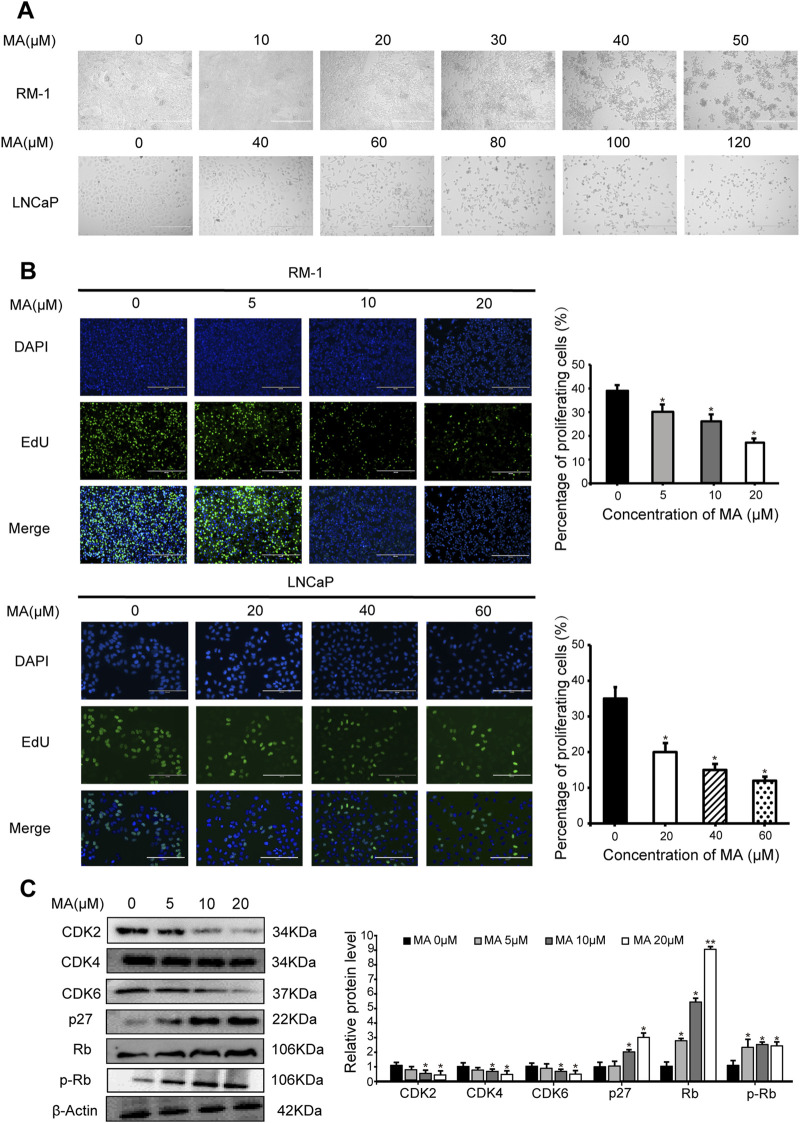
Inhibition of prostate cancer cell proliferation by maslinic acid (MA). **(A)** Cellular morphology and number of MA-treated LNCaP and RM-1 cells. Scale bar, 400 μm. **(B)** LNCaP and RM-1 cell proliferation was measured by 5-ethynyl-2′-deoxyuridine (EdU) cell proliferation assay. Scale bar, 200 μm. Quantitative analysis showing the percentage of proliferating cells. *p < 0.05 vs. control. **(C)** CDK2, CDK4, CDK6, p27, Rb and p-pRb expression in MA-treated RM-1 cells, as detected by Western blotting. Quantitative analysis showing relative protein expression levels. *p < 0.05 vs. control, **p < 0.01 vs. control.

MA exhibited dose-dependent cytotoxic activity. As the concentration of MA increased, prostate cancer cell (i.e., LNCaP and RM-1) morphology changed, cell shrinkage was observed, and adherent cells gradually detached and died (Figure 1A). MTT assay results revealed that the IC_50_ values for LNCaP and RM-1 cells treated with MA for 24 h were 88 μM and 43 μM, respectively. A sharp increase in cell death was observed above the IC_50_ value; thus, further studies were conducted with MA concentration below the IC_50_ value. The changes in cell cycle stages induced by MA were assessed using the EdU cell proliferation assay. Compared to control cells, MA treatment reduced the S-phase population of LNCaP and RM-1 cells (Figure 1B). Western blotting was performed to determine changes in the expression levels of cell cycle-related proteins in RM-1 cells treated with 0, 5, 10, and 20 μM MA. Treatment with 5–20 μM of MA augmented the expression of p27, Rb and p-Rb (Figure 1C), however, decreased that of CDK2, CDK4, and CDK6 (Figure 1C). Based on these observations, MA induced cell death by blocking G1/S transition in the cell cycle of prostate cancer cells.

### 3.2 Prostate cancer cell autophagy

**FIGURE 2:**
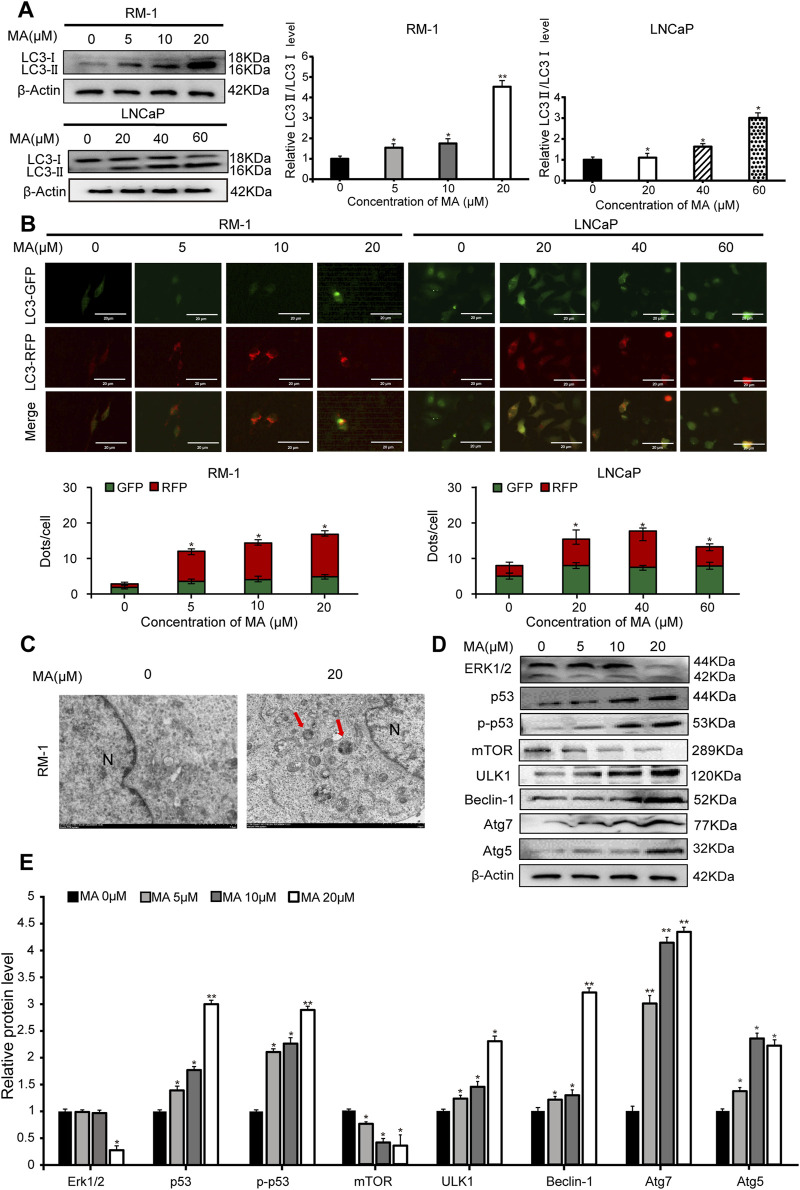
Prostate cancer cell autophagy. **(A)** Effects of maslinic acid (MA) on LC3-I/II conversion in LNCaP and RM-1 cells, as detected by Western blotting. *p < 0.05 vs. control, **p < 0.01 vs. control. **(B)** LNCaP and RM-1 cells were transfected with mRFP-GFP-LC3. Autophagic flux was detected by inverted fluorescent microscopy. Scale bar, 20 μm *p < 0.05 vs. control **(C)** Visualization of autophagosomes by transmission electron microscopy. Scale bar, 2.0 μm. **(D)** ERK1/2, p53, p-p53, Beclin1, Atg7, and Atg5 expression was detected by Western blotting. **(E)** Quantitative analysis to assess relative protein expression levels. *p < 0.05 vs. control, **p < 0.01 vs. control.

Western blotting was performed to analyze the expression levels of LC3, a biomarker of autophagy. LC3-II expression showed a significant increase in MA-treated LNCaP and RM-1 cells (Figure 2A), indicating that MA induces the formation of autophagosomes. To further validate the autophagic effect induced by MA, autophagic flux analysis was conducted. LNCaP and RM-1 cells were transfected with mRFP-GFP-LC3. The results showed enhanced red fluorescence, suggesting the activation of autophagy (Figure 2B). Further, to examine the effects of MA on autophagic flux in RM-1 cells, electron microscopy was performed to detect intracellular autophagosomes. Treating RM-1 cells with 20 μM MA for 24 h increased the number of autophagosomes in the cytoplasm (Figure 2C). Western blotting was performed to assess the expression of autophagy-related proteins in MA-treated RM-1 cells (Figures 2D,E), which revealed significantly reduced expression of ERK1/2 and mTOR and significantly increased expression of p53,p-p53, ULK1, Beclin1, Atg7, and Atg5. It indicated that the autophagy reaction mechanism was initiated after mTOR was inhibited. Altogether, these findings indicated that MA induces RM-1 cell autophagy through mTOR pathways.

### 3.3 RM-1 cell ferroptosis by MA

**FIGURE 3:**
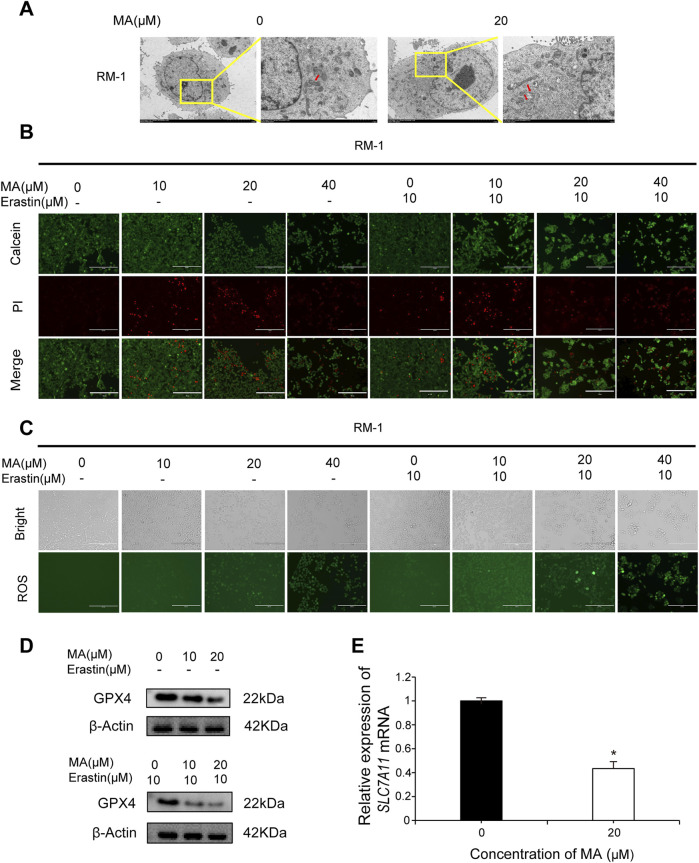
Maslinic acid (MA) induced RM-1 cell ferroptosis. **(A)** Observation of mitochondria by transmission electron microscopy. Scale bar, 2.0 μm. Red arrows indicate damaged mitochondria. **(B)** Fluorescence microscopy images of RM-1 cells co-stained with calcein (green, live cells) and PI (red, dead cells). Scale bar, 200 μm. **(C)** Reactive oxygen species (ROS) generation in RM-1 cells following MA treatment. Scale bar, 200 μm. **(D)** GPX4 expression was detected by Western blotting. **(E)** *SLC7A11* expression was detected by real-time quantitative PCR. *p < 0.05 vs. control.

Observing autophagosomes by transmission electron microscopy revealed that in comparison to control cells, mitochondrial circumference and area were reduced in MA-treated RM-1 cells (Figure 3A), indicative of a potential association between MA and RM-1 cell ferroptosis. Calcein/PI fluorescent staining was used to detect dead and living cells. MA-treated cells exhibited increased red fluorescence compared to control cells, indicating a higher number of dead cells (Figure 3B). Furthermore, treating RM-1 cells with various concentrations of Erastin enhanced MA-induced ferroptosis (Figure 3B). To measure ROS levels in RM-1 cells, we performed staining with a fluorescent probe, namely 2′-7′-dichlorodihydrofluorescein diacetate, which revealed significant ROS accumulation following MA treatment. This effect was further amplified by Erastin (Figure 3C). Western blotting indicated that MA treatment downregulated GPX4 expression (Figure 3D), and RT-qPCR showed that MA treatment downregulated *SLC7A11* expression (Figure 3E). Collectively, these results suggested that MA induces RM-1 cell ferroptosis *in vitro*.

### 3.4 Correlation analysis of transcriptome and metabolome data

**FIGURE 4:**
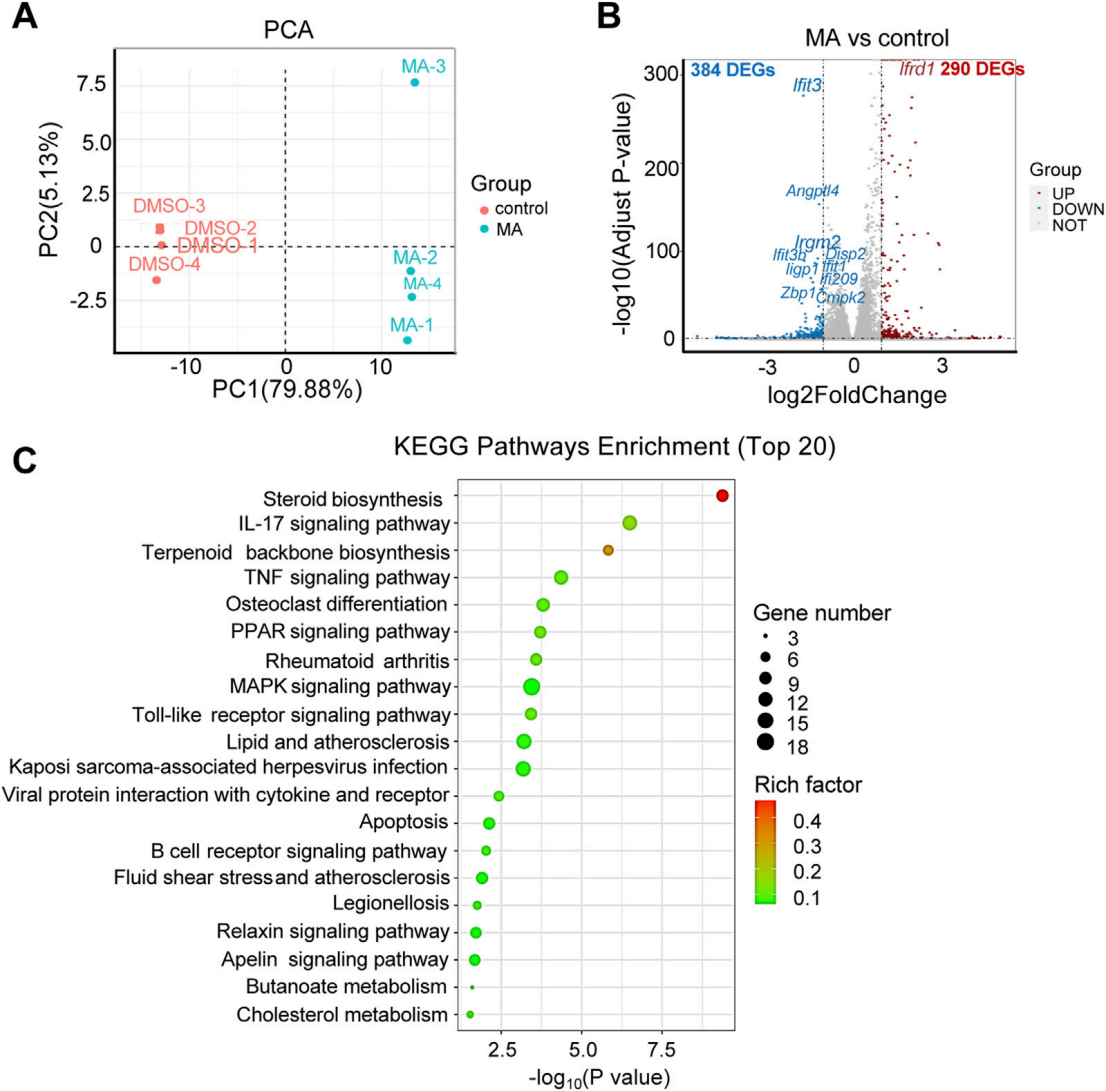
Effects of maslinic acid (MA) on RM-1 cell transcriptome. **(A)** Principal component analysis (PCA) plot of the MA-treated vs. control groups. **(B)** Volcano plot of significant differentially expressed genes (DEGs) in the MA-treated vs. control groups. Red dots indicate up- and blue dots indicate downregulated genes. **(C)** Kyoto Encyclopedia of Genes and Genomes (KEGG) pathway enrichment analysis of DEGs.

RNA-seq was performed to investigate the functional mechanisms of MA. Principal component analysis (PCA) revealed significant differences between the MA-treated and control groups Figure 4A). Differential expression analysis identified 674 DEGs (290 up- and 384 downregulated) in the MA-treated group compared to the control (Figure 4B; Supplementary Table S1). KEGG pathway enrichment analysis revealed that these DEGs were closely associated with steroid biosynthesis, peroxisome proliferator-activated receptor signaling pathway, and mitogen-activated protein kinase signaling pathway (Figure 4C).

**FIGURE 5:**
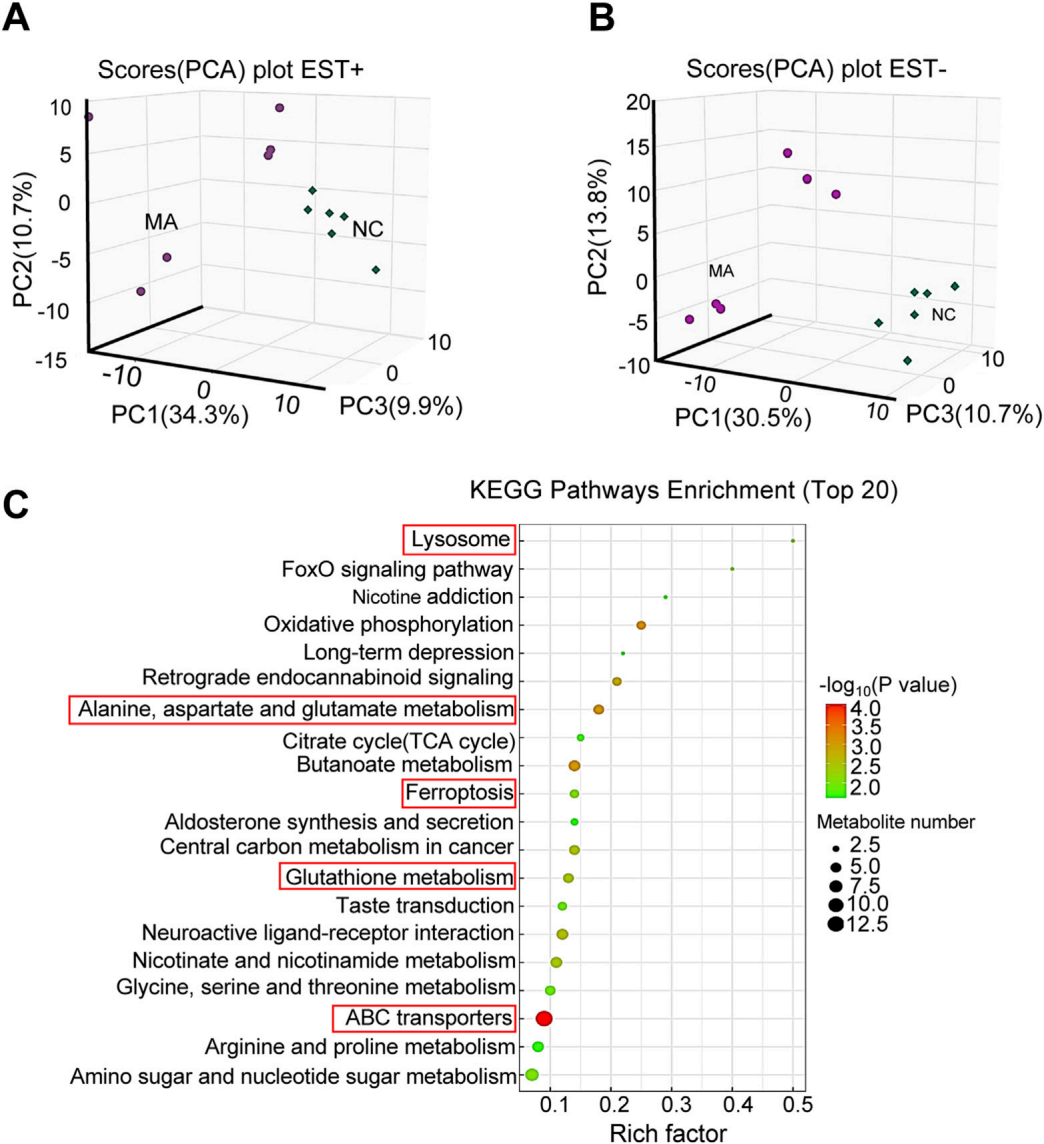
Effects of maslinic acid (MA) on RM-1 cell metabolome. Principal component analysis (PCA) plot in positive **(A)** and negative **(B)** modes. **(C)** Kyoto Encyclopedia of Genes and Genomes (KEGG) pathway enrichment analysis of differential metabolites.

With regard to metabolites, PCA showed significant differences in both positive and negative modes (Figures 5A, B). A total of 232 differential metabolites [121 in positive (Supplementary Table S2) and 111 in negative (Supplementary Table S3) modes] were identified in the MA-treated group compared to the control. KEGG pathway enrichment analysis showed that these differential metabolites were mainly enriched in alanine, aspartate, and Glu metabolism; ferroptosis; and GSH metabolism (Figure 5C).

**FIGURE 6:**
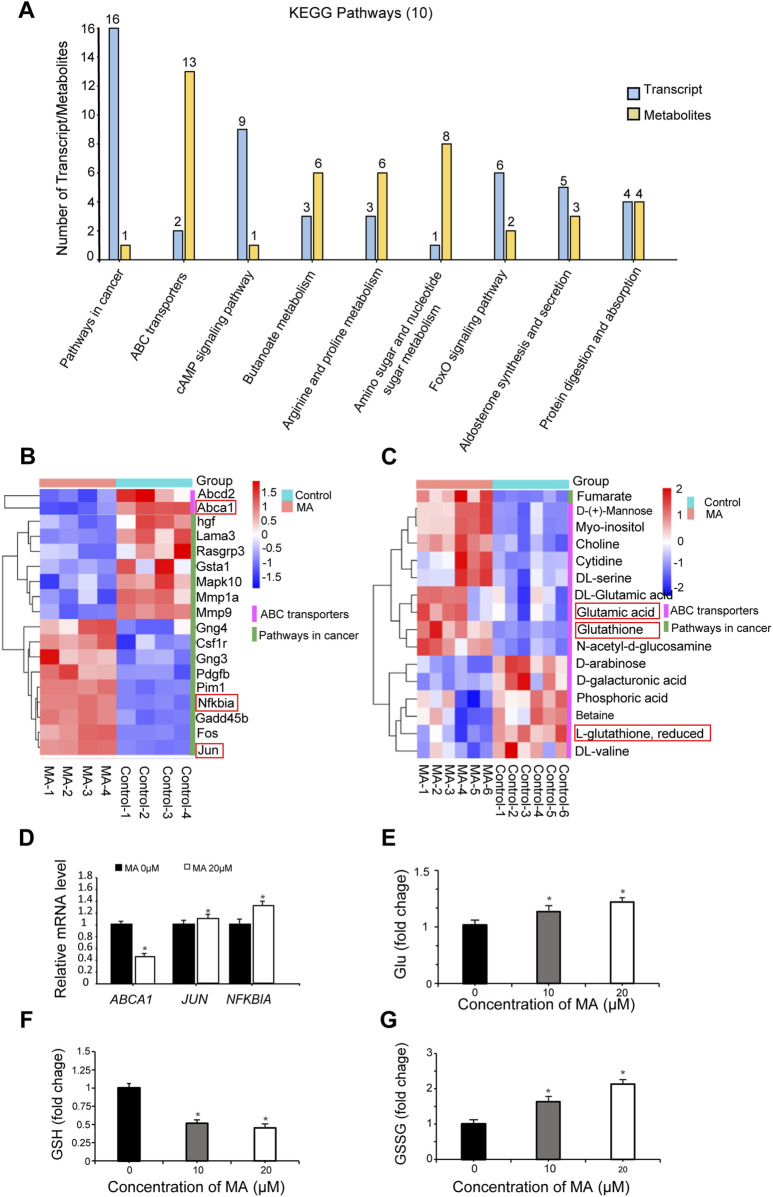
Integrated transcriptome and metabolome analysis. **(A)** Top 10 Kyoto Encyclopedia of Genes and Genomes (KEGG) pathways identified by integrated transcriptome and metabolome analysis. Heatmap of differentially expressed genes (DEGs) **(B)** and differential metabolites **(C)** in pathways in cancer and ABC transporters. **(D)** *ABCA1*, *JUN*, and *NFKBIA* expression was determined by real-time quantitative PCR. *p < 0.05 vs. control. Relative glutamate (Glu) **(E)**, glutathione (GSH) **(F)**, and oxidized GSH (GSSG) **(G)** levels were measured in RM-1 cells. *p < 0.05 vs. control.

Integrated transcriptome and metabolome analysis identified 10 crucial KEGG pathways, including pathways in cancer and ABC transporters (Figure 6A). A heatmap was plotted to depict DEGs and differential metabolites in such pathways (Figures 6B, C). The mRNA expression levels of ATP-binding cassette, sub-family A, member 1 (*ABCA1*), jun proto-oncogene (*JUN*), and nuclear factor of kappa light polypeptide gene enhancer in B cells inhibitor, alpha (*NFKBIA*), which were differentially expressed in pathways in cancer and ABC transporters following MA treatment, were verified by RT-qPCR. RT-qPCR results showed that MA inhibited the expression of *ABCA1* and promoted the expression of *JUN* and *NFKBIA* (Figure 6D), consistent with the transcriptome results. Moreover, MA treatment led to the accumulation of Glu (Figure 6E), decreased the concentration of GSH (Figure 6F), and increased the concentration of GSSG (Figure 6G) in RM-1 cells. These results suggested that MA promotes the conversion of GSH to GSSG and affects the metabolic reprogramming of GSH.

### 3.5 Tumor growth suppression by MA *in vivo*

**FIGURE 7:**
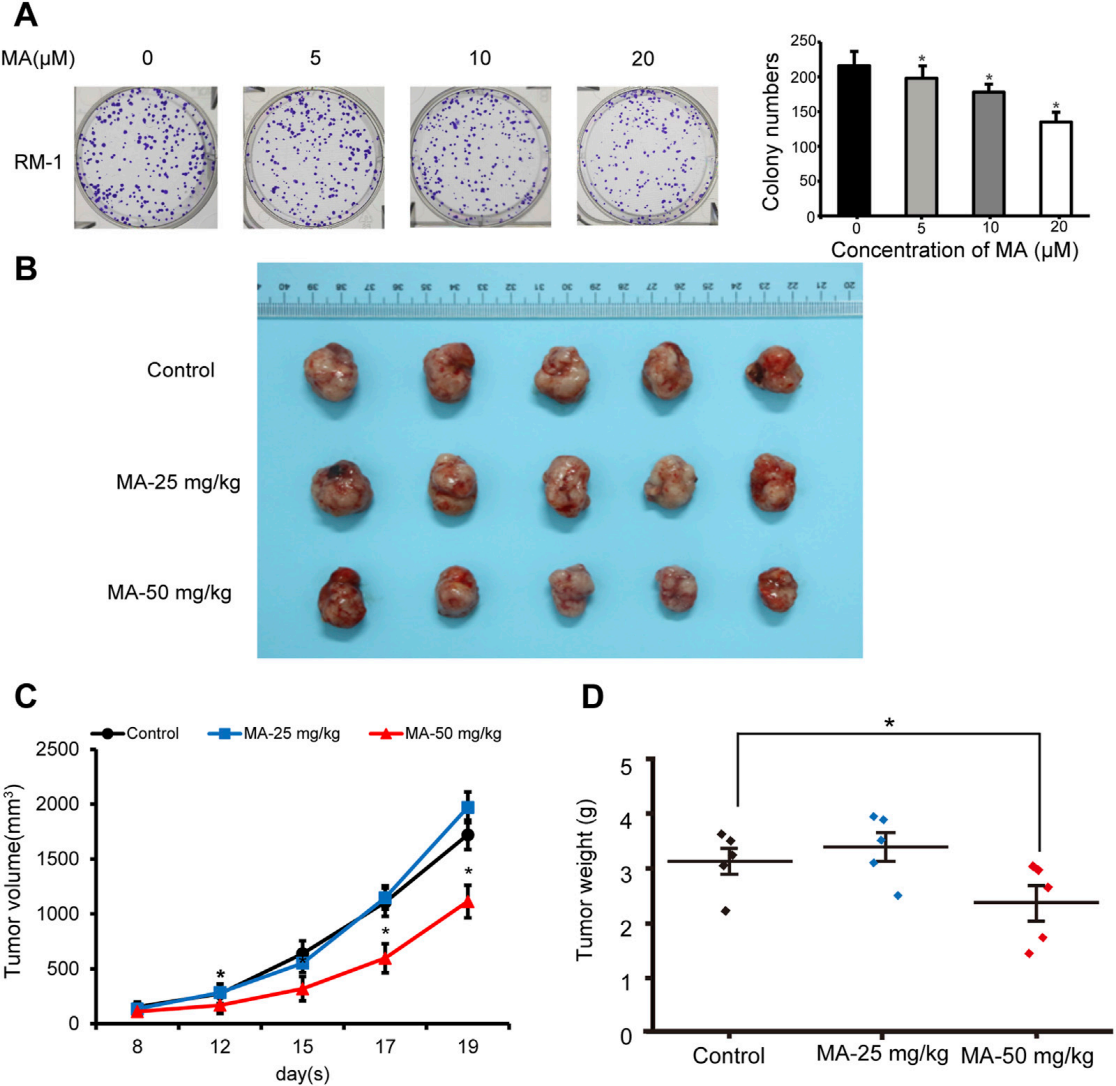
Inhibition of RM-1 cell growth by maslinic acid (MA) in a mouse model. **(A)** Effects of MA on the colony formation ability of RM-1 cells. *p < 0.05 vs. control. **(B)** Images of tumors at the end of the experiment. **(C)** Tumor volume (mm^3^) was measured to draw tumor growth curves, and **(D)** tumor weight (mg) was measured to evaluate tumor growth. *p < 0.05 vs. control.

After realizing that MA inhibits RM-1 cell growth *in vitro*, we explored its role *in vivo*. Colony formation assays were performed to evaluate RM-1 cell tumorigenicity *in vivo*. RM-1 cells were found to form colonies *in vitro*. MA inhibited this colony formation ability of RM-1 cells (Figure 7A). To further determine the anticancer effects of MA *in vivo*, a mouse model was established using RM-1 cells. Compared with the control group, the body weight of mice has no significant difference, and 50 mg/kg MA significantly inhibited tumor growth (Figure 7B), which was evidenced by a decrease in average tumor volume and weight. This indicated that MA inhibits tumor growth *in vivo* (Figures 7C, D).

## 4 Discussion

Natural products have garnered increasing attention in the field of anticancer research due to their diverse sources, stable properties, and high safety profiles. MA, a natural secondary metabolite, has been shown to act as a tumor suppressor by reducing tumor drug resistance (Wang et al., 2020), inducing DNA damage (Lu et al., 2020), inhibiting cancer cell proliferation (Jain and Grover, 2020), and inducing cancer cell apoptosis (Wang et al., 2017; Zhang et al., 2014). Herein we found MA to inhibit prostate cancer cell viability and cell cycle progression. Previous studies have reported that MA significantly enhances tumor necrosis factor-α-induced inhibition of pancreatic cancer cell proliferation by suppressing cyclin D1, COX-2, and c-Myc expression levels (Li et al., 2010). Besides, MA-induced Raji cell cycle arrest is reportedly mediated by inhibiting cyclin D1 expression and enhancing p21 protein levels (Yap et al., 2012). In this study, we found that MA inhibits the expression of CDK2, CDK4, and CDK6 and enhances that of p27, Rb and p-Rb to block G1/S transition in the cell cycle of prostate cancer cells.

Autophagy is a cellular lysosomal degradation pathway crucial for regulating cell survival and death to maintain cellular homeostasis. mTOR, a key regulator of autophagy, is activated by Akt and mitogen-activated protein kinase signaling pathways and inhibited by AMP-activated protein kinase and p53 signaling pathways. Tian et al. reported that MA induces autophagy by downregulating HSPA8 expression in pancreatic cancer cells (Panc-28) (Tian et al., 2018). Further, MA reportedly blocks the interaction between Bcl2 and Beclin1 to enhance PC12 cell autophagy (Huang et al., 2011). Jeong et al. proposed that MA serves as a potential therapeutic agent against particulate matter-induced lung injury by regulating mTOR-autophagy pathways (Jeong et al., 2020). Our study corroborated these findings, demonstrating that MA induces autophagy in prostate cancer cells via the mTOR signaling pathway.

Ferroptosis, a new type of cell death induced by lipid peroxidation, is closely linked to GSH, which is synthesized by glutamic acid, cysteine, and glycine and plays a key role in eliminating lipid ROS accumulation. As a functional subunit of the Xc-system, SLC7A11 participates in the extracellular uptake of cystine and the release of glutamate, thereby promoting GSH synthesis (Hu et al., 2022). We found that MA treatment inhibited *SLC7A1*1 activity in RM-1 cells, leading to intracellular Glu accumulation and impaired GSH synthesis.

Increasing evidence suggests that autophagy can influence ferroptosis under certain conditions (Liu et al., 2020). p53 is a critical regulator of both autophagy and ferroptosis. On one hand, p53 activation can block autophagy in response to nutritional starvation or inhibit mTOR (Kang et al., 2019). On the other hand, p53 can inhibit the transcriptional activity of SLC7A11 (Kang et al., 2019). In cancer cells, the loss of p53 function leads to decreased autophagy and increased resistance to ferroptosis (Lee et al., 2023). Herein we found that MA increased p53 expression, indicating that p53 may play a certain role in MA regulation of RM-1 cell proliferation and autophagy.

MA markedly inhibited the migration, invasion and adhesion of DU145 prostate cancer cells (an androgen-insensitive cell line), which may be mediated by hypoxia-inducible factor-1a signalling (Park et al., 2013). Our study further found that MA can also inhibit the proliferation, induce ferroptosis and autophagy of LNCaP cells (an androgen-responsive cell line). Androgen deprivation therapy (ADT) is the current gold standard for treating PCa. However, after an initial response, androgen-insensitive clones can appear, which results in cancer progression and metastasis with high mortality (Sharma et al., 2010). MA is expected to be used in combination with ADT for the treatment of AR-positive prostate cancer patients.

C57/B6 mice have good immune responses and stable genetic backgrounds, making them suitable for immunological research. Previous studies have reported that 4-1BBL-expressing tumor vaccine in combination with CTLA-4 blockade was effective in reducing tumor incidence and increasing in the survival of C57BL/6 mice transplanted subcutaneously with prostate cancer RM-1 cells (Youlin et al., 2012). Tanya B Dorff et al. report results from a phase 1, first-in-human study of prostate stem cell antigen (PSCA)-directed chimeric antigen receptor (CAR) T cells in men with metastatic castration-resistant prostate cancer (mCRPC), which evaluated the safety and biological activity of PSCA-CAR T cells in mCRPC patients (Dorff et al., 2024). Our study found that MA significantly inhibited tumor growth, which was constructed by inoculation of RM-1 cells into C57/B6 mice. This study may be helpful for the combined research and application of MA and prostate cancer immunotherapy in the later stage.

## 5 Conclusion

**FIGURE 8:**
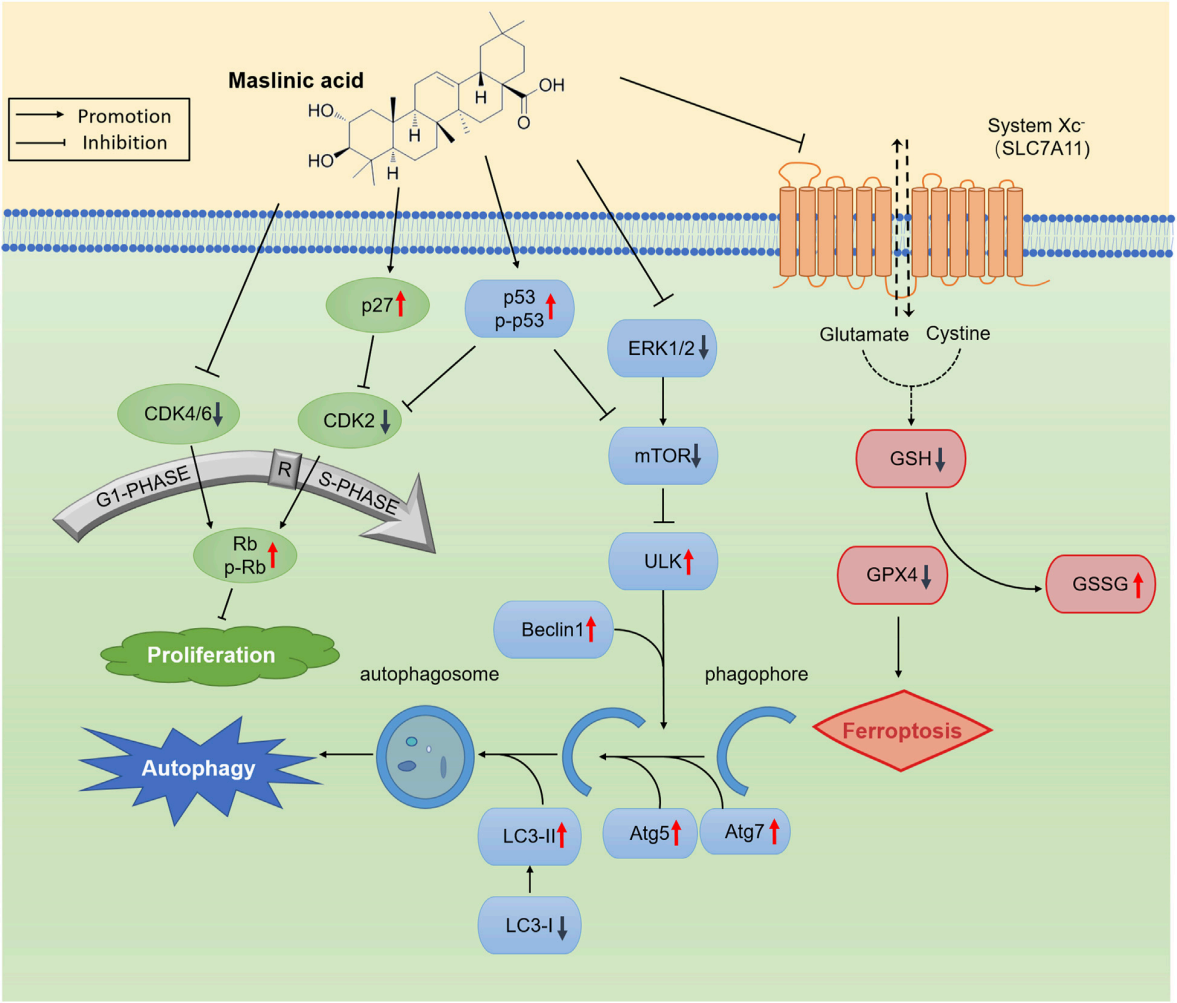
Schematic representation of the proposed anticancer mechanism of maslinic acid (MA).

To summarize, we report that MA induces autophagy and ferroptosis in prostate cancer cells (Figure 8), along with inhibiting tumor growth *in vivo*. MA exerts its antitumor effects through transcriptomic and metabolomic reprogramming, highlighting its multifaceted mechanism of action against prostate cancer. We believe that MA can serve as a promising drug for prostate cancer treatment.

## Data Availability

The original contributions presented in the study are publicly available. This data can be found here: SRA repository, accession number PRJNA1180480; MetaboLights repository, accession number MTBLS11530.
